# Physiological demands of singing for lung health compared with treadmill walking

**DOI:** 10.1136/bmjresp-2021-000959

**Published:** 2021-05-27

**Authors:** Keir EJ Philip, Adam Lewis, Sara C Buttery, Colm McCabe, Bishman Manivannan, Daisy Fancourt, Christopher M Orton, Michael I Polkey, Nicholas S Hopkinson

**Affiliations:** 1 National Heart and Lung Institute, Imperial College London, London, UK; 2 NIHR Imperial Biomedical Research Centre, London, UK; 3 Respiratory Medicine, Royal Brompton and Harefield hospitals, London, UK; 4 Health Sciences, Brunel University London, London, UK; 5 Department of Behavioural Science and Health, University College London, London, UK

**Keywords:** exercise, COVID-19, lung physiology, pulmonary rehabilitation

## Abstract

**Introduction:**

Participating in singing is considered to have a range of social and psychological benefits. However, the physiological demands of singing and its intensity as a physical activity are not well understood.

**Methods:**

We compared cardiorespiratory parameters while completing components of Singing for Lung Health sessions, with treadmill walking at differing speeds (2, 4 and 6 km/hour).

**Results:**

Eight healthy adults were included, none of whom reported regular participation in formal singing activities. Singing induced acute physiological responses that were consistent with moderate intensity activity (metabolic equivalents: median 4.12, IQR 2.72–4.78), with oxygen consumption, heart rate and volume per breath above those seen walking at 4 km/hour. Minute ventilation was higher during singing (median 22.42 L/min, IQR 16.83–30.54) than at rest (11 L/min, 9–13), lower than 6 km/hour walking (30.35 L/min, 26.94–41.11), but not statistically different from 2 km/hour (18.77 L/min, 16.89–21.35) or 4 km/hour (23.27 L/min, 20.09–26.37) walking.

**Conclusions:**

Our findings suggest the acute metabolic demands of singing are comparable with walking at a moderately brisk pace, hence, physical effects may contribute to the health and well-being benefits attributed to singing participation. However, if physical training benefits result remains uncertain. Further research including different singing styles, singers and physical performance impacts when used as a training modality is encouraged.

**Trial registration number:**

ClinicalTrials.gov registry (NCT04121351).

**Key messages**

- How physiologically demanding is singing compared with treadmill walking?

- The acute physiological demands of singing are comparable with walking at a moderately brisk pace.

- To our knowledge, this is the first study to compare the physiological demands of singing with physical activity. Given the need to find enjoyable and well-tolerated physical activities to promote health and well-being, these are important preliminary findings.

## Introduction

Singing is an ubiquitous cultural practice throughout history and across the world,^1^ and participation in singing is believed to have a range of health and well-being benefits.^2 3^ Research to date has predominantly focused on psychosocial and psychobiological impacts.^4–8^ However, the cardiorespiratory demands of singing, and the potential for it to serve as a form of exercise and contribute to daily physical activity, are less well examined.

An appreciation of the physiological demands of singing could improve understanding of how best to use singing in a therapeutic capacity. An example of a structured therapeutic singing intervention is Singing for Lung Health (SLH), which has been developed as a strategy to help people with respiratory disease,^8–12^ particularly those who continue to be limited by breathlessness despite optimal medical care.^13–15^ Though high-quality research on the impacts of SLH is limited,^16^ participants report a range of biopsychosocial impacts,^8 12^ including physical improvements relating to balance^17^ and physical aspects of quality of life.^8^ The popularity of SLH for people with respiratory disease continues to grow. Around 100 groups exist in the UK, with many more internationally,^18–20^ and now also online.^17^ Such approaches are potentially deliverable at relatively low financial and resource costs through using existing social and cultural capital, and as such, ongoing work suggests a great deal of potential for these approaches in low-resource settings.^20 21^ Furthermore, it is known that exercise training is one of the most effective management strategies for people with long-term respiratory conditions,^22^ usually in the form of pulmonary rehabilitation (PR), however many people are unable to access PR,^23^ or do not want to do it, hence alternative approaches could be complementary in expanding provision of exercise training opportunities and diversifying delivery modalities, if an evidence base were to be established.

Additionally, identifying existing, enjoyable and well-attended physical activities of sufficient intensity to be considered exercise is useful from a public health and health promotion perspective. Physical activity is important both to maintain health and to mitigate the impact of long-term medical conditions.^24^ This is particularly relevant during the present COVID-19 pandemic, where physical distancing measures to reduce risk of COVID-19 transmission, combined with the concerns about the virus itself, are having unintended negative impacts including inactivity, social isolation and anxiety.^25 26^ As such, there is an urgent need to provide and support evidence-based strategies that are deliverable in the current situation and beyond, which could, for example, include online singing groups.^17 27^

To evaluate this further, we undertook a study to compare cardiorespiratory parameters during singing, and various SLH exercises, with (1) rest and (2) three different walking speeds.

## Methods

### Participants

We conducted a non-blinded observational study. A convenience sample of colleagues and staff at the National Heart and Lung Institute were approached face-to-face and invited to participate in the study. The initial intention was to recruit 12 participants, which was felt to be a reasonable size to explore the research questions based on previous physiological studies conducted by the research team and reviewing relevant literature. However, the implementation of restrictions on potentially aerosol-generating procedures due to the COVID-19 pandemic meant we decided to stop at eight. None of the participants sang regularly. Inclusion criteria included: age 18–99 years; no significant medical conditions or active musculoskeletal disease impairing exercise; no contraindications to exercise or spirometry as per American Thoracic Society/European Respiratory Society (ATS/ERS) criteria; and capacity to consent to exercise testing.

### Physiological parameter assessment

Physiological parameters assessed were oxygen consumption (VO_2_) mL/kg/min, end tidal carbon dioxide (CO_2_) (kPa), heart rate (beats per minute), minute ventilation (L/min), respiratory rate (breaths/min) and mean volume per breath (L/breath). Gas analysis and flow were collected using JLab software package, Breath-by-Breath, and the Jaeger Oxycon Pro and Vyaire Oxycon mobile devices depending on availability. The device was calibrated between participants as per the manufacturer’s instructions provided with the device. Heart rate was assessed using the Polar heart rate monitor (Polar, Finland). Measures of perceived effort and dyspnoea were recorded at baseline and following each component according to the Borg Rating of Perceived Exertion (RPE)^28^ and Borg Modified Dyspnoea^29^ Scales. Each stage of the protocol was completed for 2 min with 20 s between each section to allow for a verbal reminder of the next stage of the protocol to the participant, equipment check and change of participant position if necessary. The 2 min duration of protocol components was selected based on a compromise between recommendations regarding exercise testing guidelines,^30^ being representative of real-world SLH sessions, and pilot work comparing the second minute values with longer protocol duration, which suggested stability of values during the second minute of each component. As such, the mean value from the second minute of assessment was used. Data were recorded continuously as the protocol was completed by each participant.

Spirometry was conducted as per ATS/ERS Guidelines^31^ by KEJP (respiratory registrar) and AL (respiratory physiotherapist) who are both trained and experienced in these tests. Physical activity intensity was considered as light, moderate and vigorous, according to metabolic equivalents (METs), derived from the VO_2_ mL/kg/min data, with light physical activity if below 3 METs, moderate if between 3 and 6 METs, and vigorous if above 6 METs.^32^ METs for each component were calculated by dividing by 3.95 mL/kg/min, which was the median measurement for the group during the resting phase 1.

### Singing protocol

SLH is a structured group singing programme for people with chronic respiratory conditions^8^ (see https://www.blf.org.uk/support-for-you/singing-for-lung-health). The components of an SLH session are similar to those found in most community choirs and singing groups, but in addition, with the aim of improving participants’ symptoms through song, breathing exercises and relaxation techniques. Components were selected from SLH because it is an established method of group singing for which the session content has been clearly defined and evaluated.^8 11^ Each component was demonstrated by AL to each participant who briefly practised the content of each component to show understanding, before resting for 30 min during study set-up.

**Figure 1:**
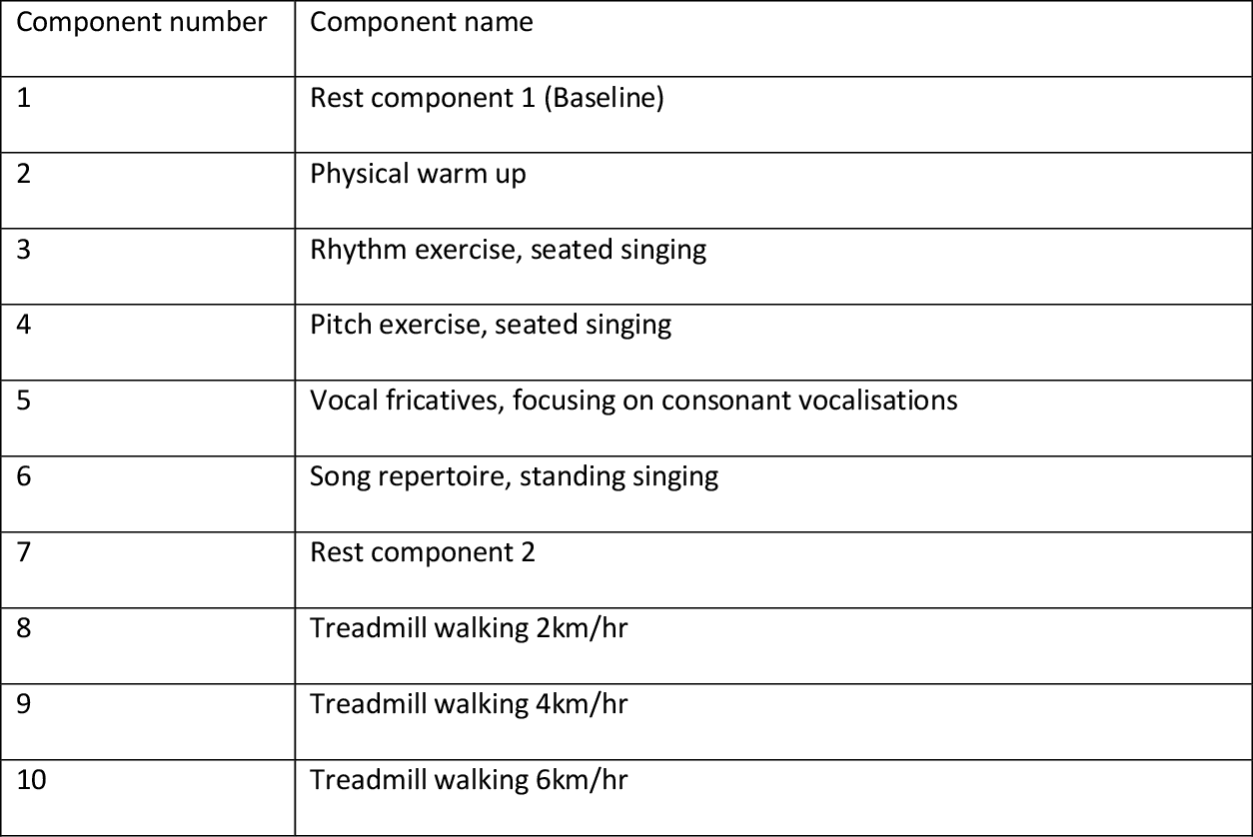
Protocol in brief.

Participants completed the following protocol with components completed sequentially from 1 to 10, with each component lasting 2 min. The full study protocol is provided in the online supplemental material. However, components in brief were as follows (figure 1):

10.1136/bmjresp-2021-000959.supp1 Supplementary data

Of note, the singing/vocalising components of the study are undertaken with additional physical actions. This would be commonly seen in community choir singing, however, should also be noted when interpreting results. Full details of the movements undertaken during each component are provided in the online supplemental file (bit.ly/3fdnEax).

Walking speeds were selected as being representative of a slow, medium and fast walk. These speeds also cover the National Health Service definition of a ‘brisk’ walk of 3 miles/hour (4.8 km/hour),^33^ recommended as moderate intensity exercise which can increase aerobic fitness.^34^

Two rest components were included to assess if the protocol included sufficient time for full recovery between components. This was done by including ‘rest component 2’ after the component 6 song repertoire’ which was expected to be the most physiologically demanding. Parameters from ‘rest component 2’ could then be compared with ‘rest component 1 (baseline)’ to see if they normalised. Additionally, ‘rest component 2’ was placed after completion of the vocalising components, to enable participants physiological parameters to return to baseline before the walking components.

### Statistical analysis

Analyses were carried out using Stata V.14 (StataCorp, Texas, USA). The Friedman test was used to assess for differences in the impact of protocol components on physiological parameters. Post-hoc Wilcoxon signed-rank tests were used for pairwise comparisons between singing, rest and walking. A non-parametric test, rather than a parametric test, was used due to the small sample size. Statistical significance was set at p<0.05. Readers wanting to adjust for multiple comparisons within each physiological parameter could apply a Bonferroni alpha of 0.013 (p<0.05 divided by four tests per parameter). Further adjustment for multiple comparisons across the different physiological parameters was not calculated given our sample size was small and our study exploratory. Data are presented to two significant figures.

### Patient and public involvement

The idea for the study came from discussion between the study authors (KEJP and AL) with SLH participants who previously attended a face-to-face SLH group that took place in the Royal Brompton Hospital, prior to COVID-19 restrictions. Between 8 and 12 people regularly attended these sessions. The study design was further discussed with four expert patients in a patient and public involvement research group that regularly takes place at the Royal Brompton Hospital, who highlighted potential physical benefits related to SLH participation and support for exploring this topic. Our research proposal was well received as there was clear interest in improving our understanding of how such approaches might impact health and well-being.

## Results

Participant characteristics are shown in table 1. Data comparing physiological parameters during singing with rest and walking at three different speeds are shown in table 2. Friedman tests demonstrated that the protocol components induced differences in all physiological parameters: VO_2_ mL/kg/min (Q (9)=65.78, p<0.001); METs (Q (9)=65.78, p<0.001); end tidal CO_2_ (Q (9)=45.19, p<0.001); heart rate (Q (9)=58.44, p<0.001); minute ventilation (Q (9)=57.30, p<0.001); respiratory rate (Q (9)=48.60, p<0.001); volume per breath (Q (9)=43.31, p<0.001); Borg Breathlessness Scale (Q (9)=32.91, p<0.001); Borg RPE Scale (Q (9)=40.50, p<0.001).

**Table 1:** Participant characteristics

Demographic	Mean (SD)
Age (years)	32 (4)
Gender	2 female, 6 male
Height (m)	1.71 (0.07)
Weight (kg)	77.1, (15.6)
Ethnicity	4× white European; 4× Arabic (3× Saudi, 1× Egyptian)
BMI	26.4 (5.8)
FEV_1_ (L)	3.81 (1.01)
FEV_1_ % predicted	95.9 (17.2)
FVC (L)	4.86 (1.09)
FVC % predicted	102.5 (14.3)

BMI, body mass index; FEV_1_, forced expiratory volume in 1 s; FVC, forced vital capacity.

**Table 2:** Comparison of singing with rest and walking at three different speeds

Cardiorespiratory parameter	Singing repertoire (component 6) median (IQR)	Baseline rest period 1	Difference from singing repertoire (p value*)	Walking at 2 km/hour median (IQR)	Difference from singing repertoire (p value*)	Walking at 4 km/hour median (IQR)	Difference from singing repertoire (p value*)	Walking at 6 km/hour median (IQR)	Difference from singing repertoire (p value*)
		Median (IQR)							
VO_2_ mL/kg/min	16.27 (10.74–18.86)	3.95 (3.69–4.35)	−12.32 (0.012)	8.19 (7.26–9.01)	−8.08 (0.012)	10.42 (9.68–11.33)	−5.85 (0.036)	15.39 (14.68–16.64)	−0.88 (1.00)
METs	4.12 (2.72–4.78)	1.00 (0.93–1.10)	−3.12 (0.012)	2.07 (1.84–2.28)	−2.05 (0.012)	2.64 (2.45–2.87)	−1.48 (0.036)	3.90 (3.72–4.21)	−0.22 (1.00)
										End tidal CO_2_ kPa	5.16 (4.91–5.51)	4.24 (3.80–4.52)	−0.92 (<0.05)	4.43 (3.88–4.45)	−0.73 (<0.05)	4.62 (4.08–4.91)	−0.54 (0.069)	4.91 (4.40–5.14)	−0.25 (0.12)
Heart rate (bpm)	108 (97–114)	76 (63–82)	−31 (0.012)	86 (81–91)	−22 (0.012)	99 (88–107)	−9 (0.042)	108 (101–119)	0 (0.62)
Minute ventilation (L/min)	22.42 (16.83–30.54)	11 (9–13)	−10.9 (0.012)	18.77 (16.89–21.35)	−3.72 (0.069)	23.27 (20.09–26.37)	+0.85 (0.89)	30.35 (26.94–41.11)	+7.93 (0.017)
Respiratory rate (breaths/min)	10 (7–13)	15 (14–17)	+5.35 (0.017)	23 (21–29)	+12.75 (0.012)	23 (20–31)	+12.49 (0.012)	24 (20–35)	+13.32 (0.012)
Volume per breath (L/breath)	2.11 (1.92–2.70)	0.69 (0.63–0.77)	−1.42 (0.0117)	0.80 (0.68–0.98)	−1.31 (0.012)	0.93 (0.86–1.05)	−1.18 (0.012)	1.21 (1.09–1.43)	−0.90 (0.012)
Borg Dyspnoea Scale	1.0 (1.0–2.5)	0 (0–0)	−1 (0.013)	0.00 (0.00–0.50)	−1 (0.019)	0.75 (0.50–1.00)	−0.25 (0.049)	1.00 (0.75–1.00)	0 (0.049)
Borg Rating of Perceived Exertion Scale	8.5 (8.0–9.0)	6 (6–6)	−2.5 (0.019)	6.00 (6.00–7.00)	−2.5 (0.035)	7.50 (7.00–8.00)	−1 (0.052)	9.00 (8.00–9.00)	+0.5 (0.8788)

*Wilcoxon signed-rank test. Data are provided to two decimal places, or less, when appropriate for degree of accuracy of the specific measurement.

bpm, beats per minute; CO_2_, carbon dioxide; METs, metabolic equivalents; VO_2_, oxygen consumption.

**Figure 2:**
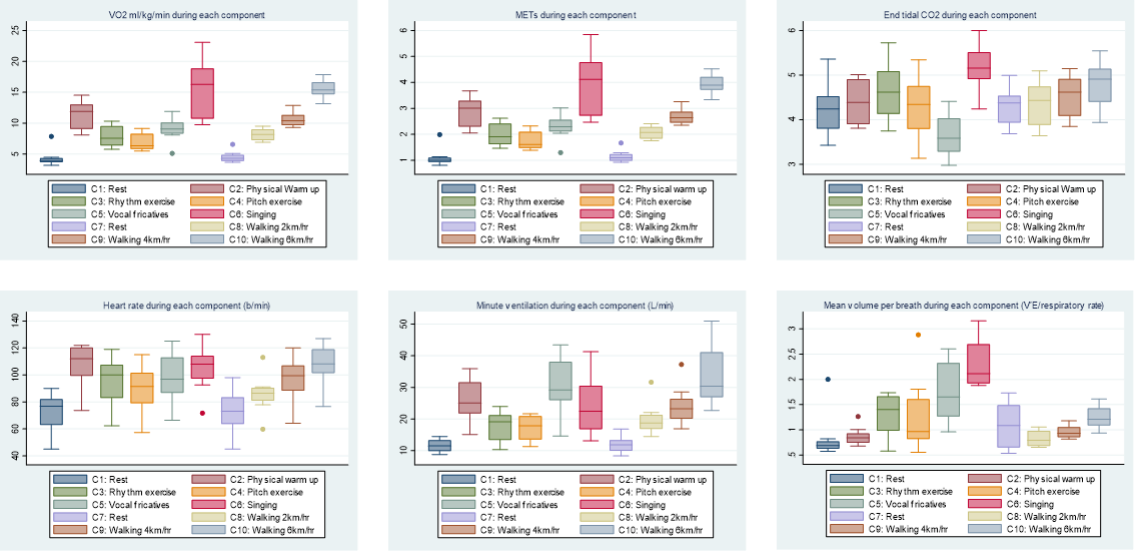
Box and whisker plots of physiological parameters during each component of the protocol. For box and whisker plots, the line in the centre of the box represents the median, the box includes the first to third quartiles, the whiskers indicate upper and lower values (excluding outliers), the dots represent possible outliers. Friedman tests demonstrated that the protocol components included differences in all physiological parameters, p<0.001. CO_2_, carbon dioxide; METs, metabolic equivalents; VE, minute ventilation; VO_2_, oxygen consumption.

Data are shown in figure 2. The main singing condition (protocol component 6) showed that singing induced statistically significant increases in VO_2_, heart rate and volume per breath compared with rest conditions, walking at 2 km/hour, or walking at 4 km/hour (pairwise comparisons using Wilcoxon signed-rank test). Minute ventilation was higher during the singing component than at rest, and lower than walking at 6 km/hour, but not statistically significantly different from walking at 2 or 4 km/hour. End tidal CO_2_ was higher in singing than at rest or walking at 2 km/hour, but not statistically different from walking at 4 or 6 km/hour. Borg Breathlessness Scale ratings suggest singing was associated with an increased sensation of breathlessness compared with rest and all walking speeds. Perceived exertion during singing was greater than during rest and walking at 2 km/hour, but not different from walking at 4 or 6 km/hour. Respiratory rate was lower during singing than rest or walking, however this is likely due to the phrasing of the songs, rather than being a representative of a physiologically driven respiratory rate.

## Discussion

### Main finding

We found that singing produced changes in physiological parameters including VO_2_, end tidal CO_2_, METs, heart rate and minute ventilation, comparable with those seen when walking at a moderate to brisk pace, consistent with the changes in these parameters seen during moderate intensity physical activity.

Research regarding the oxygen cost of singing by people with limited singing experience is limited. Sliiden *et al* present data from 20 professional musical theatre performers which suggest similar physiological responses to the current study when singing compared with rest, including heart rate, VO_2_, minute ventilation and breath volume.^35^ Another study of nine final-year musical theatre students compared cardiorespiratory parameters while singing and dancing together, with dancing alone. The study found significantly lower breathing frequency and higher lactate when singing and dancing together, compared with dancing alone, but other parameters including VO_2_ and heart rate did not differ significantly.^36^ However, singing alone (without dancing) was not compared with rest which limits comparisons. Regarding ventilatory volumes, our findings support previous research that suggests increases during singing and speech compared with spontaneous breathing.^7 37–41^ However, much of the previous research concerns speech alone, and where singing has been investigated, the studies have largely focused on professional singers, or employed limited protocols that do not fully represent the range of activities engaged in during a community singing group. As such, application of previous research findings to the most common contexts in which people sing is challenging. To our knowledge, this is the first study to systematically assess physiological parameters in people who do not sing regularly, including pulmonary ventilation volumes, during the various singing activities commonly found in amateur community singing groups. As such, our findings build on those of other studies by demonstrating comparable physiological responses related to singing in non-singers, and by comparing singing with a standardised form of physical activity in the form of treadmill walking. Of note, the relative increases above baseline in ventilatory parameters may be of importance when considering aerosol transmission of infectious agents, including SARS-CoV-2.^42^

An important consideration when interpreting our findings is that the extent to which people are moving is also likely to be a major factor in determining the physiological demands of the activity. Though completely static singing is unrealistic, we should consider that different types of singing encourage different levels of body movement, gesture and dance like movements, in addition to voice production. A further point for consideration is the extent to which changes in the physiological parameters assessed result from physical exertion, or a degree of relative hyperventilation required for vocalisation. For example, one might expect to see larger ventilatory volumes, and possibly heart rate, because of the air flow velocity and volume requirements for vocalisation. However, the pattern of end tidal CO_2_ during singing, compared with walking, suggests that hyperventilation alone does not account for the changes in the other parameters seen during the singing component. Furthermore, while minute ventilation approximately doubles from baseline, VO_2_ approximately quadruples, suggestive of an important contribution from higher cardiac output, respiratory muscle oxygen extraction and skeletal muscles involved in movement, however the relative contribution of these factors has not been investigated here.

It is also useful to consider how our findings apply to people with respiratory disease. In the current studies, participants did not have any activity-limiting illnesses and are substantially younger than many people with common long-term respiratory conditions, such as chronic obstructive pulmonary disease. People with respiratory conditions may be more restricted in their ability to engage in singing activities in general, which could influence the physiological demands experienced. However, SLH sessions are specifically designed for people with respiratory disease and personally adapted to individual participant’s abilities during sessions, to enable participation despite individual restrictions. However, the potential for physiological responses to differ by age group and the presence of respiratory disease highlights the need to evaluate potential differences in future research.

### Methodological considerations

This study has multiple strengths. To our knowledge, this is the first study to compare the physiological demands of singing with walking, using measures of ventilation, VO_2_, end tidal CO_2_, and perceived effort and dyspnoea simultaneously. The focus on people who are not professional singers or performers makes the findings highly relevant for people who do not regularly engage in singing.

Certain limitations should be mentioned. First, the use of healthy, relatively young participants may limit the extent to which our findings can be extrapolated to older people, or those with significant medical conditions, such as those with chronic respiratory disease (CRD). However, individuals with CRD are likely to find activities such as singing more, rather than less physiologically demanding, as a proportion of their VO_2_ max.^43^ Therefore, one might reasonably suspect that the potential for physical benefits related to training effects would also be increased, though in what way, and to what extent, remains unclear. Additionally, this would require the individuals with CRD to engage with the activity in the same way as the healthy volunteers of the current study, which for many people with CRD would not be possible. Given the multiple uncertainties regarding specific responses in people with CRD, further research including such participants is clearly required. Second, the sample size is small; although it was sufficient to meet the aims of the study by comparing the parameters during protocol components, replication of our findings in larger samples is encouraged. Third, although we considered real-world applicability when developing the components of the protocol, the total protocol duration was approximately 25 min, while most community singing sessions are longer. As such, further studies during real-world community singing group sessions would be of interest. Lastly, though this study has demonstrated that singing induces physiological responses that are similar in magnitude to moderate intensity physical activity, this study has not assessed training effects of singing. As such we cannot draw clear conclusions from this study alone regarding impacts on physical fitness.

It is possible that given the jaw movement required for singing that a dynamic air leak could have gone unnoticed. However, this is unlikely as we tested the fit before starting. Furthermore, if there was a leak, the ventilatory values would have been underestimated, rather than overestimated. Additionally, this would not have influenced the relative proportions of gases recorded in the analysis.

This study has raised multiple directions for future research. To build on these findings, future studies could include maximal exercise tests for comparison; evaluate if training effects occur following a programme of singing; directly compare professional and amateur singers; specifically assess the impact of musical genre, volume and physical movements; and compare healthy controls with people with certain chronic diseases, in whom singing is being delivered in a therapeutic context. It would also be valuable to explore how the different session components could be adapted and varied, and how this influences outcomes.

## Conclusion

This study demonstrated that singing when standing induced acute physiological responses similar in magnitude to moderate intensity physical activity. The study also identified increases in minute ventilation and breath volumes during singing and during singing-related activities, that may be important when considering risk of transmission of respiratory infections including SARS-CoV-2. These findings suggest that health and well-being benefits attributed to singing participation, may in part, result from physical mechanisms. Further research including different types of singing, and singers, and training effects would be valuable.

## Data Availability

Data may be made available on reasonable request.
