# Supplementary material for: Physiological demands of singing for lung health compared with treadmill walking
Source: BMJ Open Respir Res. 2021 May 27;8(1):e000959. doi: 10.1136/bmjresp-2021-000959 (PMC8162088; doi:10.1136/bmjresp-2021-000959)

Supplement file: The physiological demands of Singing for Lung Health compared to treadmill walking

Authors: Keir EJ Philip[1,4]*, Adam Lewis[2]*, Sara C Buttery[1,4], Colm McCabe[1,4], Bishman Manivannan[1], Daisy Fancourt[3], Christopher Orton[1,4], Michael I Polkey[1,4], Nicholas S Hopkinson[1,4]

*joint first author

Correspondence to Keir Philip, email: k.philip@imperial.ac.uk

Affiliations:

1) National Heart and Lung Institute, Imperial College London, London, United Kingdom
2) Department of Health Sciences, Brunel University, London, United Kingdom
3) Department of Behavioural Science and Health, University College London, London, United Kingdom
4) Royal Brompton and Harefield NHS Foundation Trust, London, United Kingdom

Supplement

1. Component learning phase

Consenting participants took part in SLH activities under the instruction of Dr Adam Lewis (AL) who teaches on the Singing for Lung Health Singing Leader training programmes and demonstrated component exercises during the set up and calibration of other equipment. These simple exercises are regularly undertaken by people with significant medical problems, so we anticipated that all participants would have been able to complete these activities. However, participants were informed they were free to stop at any point. Participants had to demonstrate, repeat, and confirm understanding of the example exercises of each component demonstrated. AL had to be satisfied they could be perform them effectively, keeping to the rhythm of AL's performance for each component. Participants were instructed that they would be led throughout the protocol by AL and able to mirror the activities shown. Participants were given the opportunity to practice and ask questions about each component Learning all the singing activities took approximately 15 minutes. All participants had previously used the treadmill equipment during other studies and so no learning phase was required for this. All the vocal exercises and song repertoire are selected to be suitable for people with no previous formal singing training, and for those with respiratory conditions. As such the pitch range, rhythmic complexity and tempo are kept within achievable limits.

The timing of each following component and instruction period was recorded by KP.

Component 1: Rest period 1, baseline assessment: Participants sat for five minutes at rest. Two minutes of resting condition was then recorded prior to taking part in six of the core components of a SLH session for periods of two minutes per component.

Component 2: Physical warm up: gentle, gestural, dance-based movements, standing, performed to music ("Sugar Sugar", by The Honeys): Participants commenced a physical warm up accompanied to music: The song was played via Adam Lewis's mobile phone. Participants followed AL through the following in sitting: Alternate toe taps, Alternate heel digs, hamstring curls under chair, alternate punching then increasing range of flexion (pretending to climb a ladder), forward leaning and sweeping the floor with both hands moving all the way to full shoulder flexion with widely spread hands (all movements should be comfortably within the participants individual range of movement). The participants were then instructed to stand and repeat in standing followed by neck rotation and side-flexion exercises. Participants returned to a sitting position at the end of the two minute period.

Component 3: Singing based rhythm exercise. Participants were instructed to follow AL through a song called 'Alive, Awake, Alert, Enthusiastic' This phrase was repeated with each word having an associated body action: Both AL and participants touched their heads with both hands for 'Alive', touched their shoulders for 'Awake', touched their knees for 'Alert' and clapped, then flex their shoulders to 90 degrees and had elbows fully extended and wrists supinated for 'enthusiastic'. These actions were repeated in song. Individuals then dropped the 'Alert' action and sung word. This was then repeated by singing 'Alert' but dropping 'Enthusiastic'. The Rhythm exercise was be performed in a sitting posture. Participants were instructed to get each repetition of 'Alive, awake, alert, enthusiastic' sung in a single breath.

Component 4: Pitch exercise: seated singing, focusing on challenging the accuracy and range of pitch achieved. Participants were instructed to follow AL singing a song with the lyrics: 'Elevator won't you take me 1,2,3,4,5. Elevator won't you take me 5,4,3,2,1' This phrase was repeated with a higher pitch being reached with each consecutive number and the pitch coming back down in scale with each descending number. Participants mirrored AL in standing, with feet slightly wider than shoulder width, with slight flexion in the knees. With each increasing number that was sung, the participant raised their left hand parallel to their xiphisternum ('1') in approximately 10cm increments until level '5' was approximately at the level of their nose. The phrase was repeated with number '3' being absent and then repeated with both number '3' and '5' being absent. All numbers were then sung again and repeated. Participants were encouraged to get the whole sung phrase out in 1 breath.

Component 5: Fricatives – a vocal exercise focusing on consonant vocalisations. Participants were asked to mirror AL through a set of voiced fricatives in a standing posture in step stance. 'Ssshhhh' 'jjjjj' and 'vvvv' were repeated with participants putting one hand on their abdomen just above the pelvic bone, and the other hand below the xiphisternum. Then alternating hand position by both hands moving to be in a fist position and pushed into their sides in between the lower ribs and hips. These hand positions were designed to provide tactile feedback from the abdominal muscle use with the exhaled breath. Participants were then asked to pulse the voiced fricatives in a '1, 2' rhythm. Finally participants were asked to repeat the voiced fricatives whilst in alternate step stance.

Component 6: Repertoire: Singing standing up. Participants sang '1 bottle of beer' acapella. The lyrics are as follows:

One bottle of beer, two bottle of beer, three bottle of beer,

four bottle of beer, five bottle of beer, six bottle of beer,

seven bottle of beer, 8 POP! (1 breath)

Fish and chips and vinegar, vinegar, vinegar,

Fish and chips and vinegar, pepper, pepper, pepper POT! (1 breath)

Oh you can't put your muck in our dustbin,

our dustbin, our dustbin,

You can't put your muck in our dustbin,

our dustbin's FULL! (1 breath)

Individuals were encouraged to repeat each segment in one exhaled breath. Individuals were asked to count using their hands and fingers for actions and sway laterally with each number with feet slightly wider than shoulder width and slightly flexed knees. This swaying motion is exaggerated with 'fish and chips…' pretending to cradle the fish and chips like a baby as if they were wrapped in newspaper. Then during the 'muck in our dustbin' participants will be asked to stamp alternate feet forward and point with alternate hands repeatedly.

Component 7: Rest component 2: This was included to i) compare with the baseline measurements, to assess if the protocol included sufficient time for full recovery between components; and ii) to enable participants to return to baseline before the walking components. This was an active rest period as it was accompanied by some relaxation prompts given by AL. Participants were instructed to sit down and follow a guided visualisation relaxation with imagery of being on a beach relaxing on a deck chair with the warm sun on the face and the perfect breeze flowing and the smooth rhythmical sound of waves in the background. Instructions regarding

optimising individual's body posture were given and any points of muscular tension that was noted by AL was addressed with guided muscle relaxation instructions such as 'releasing the jaw', and 'open your palms to the sky'.

Component 8: treadmill walking at 2km/hr, no incline

Component 9: treadmill walking at 4km/hr, no incline

Component 10: treadmill walking at 6km/hr, no incline

Additional Graph

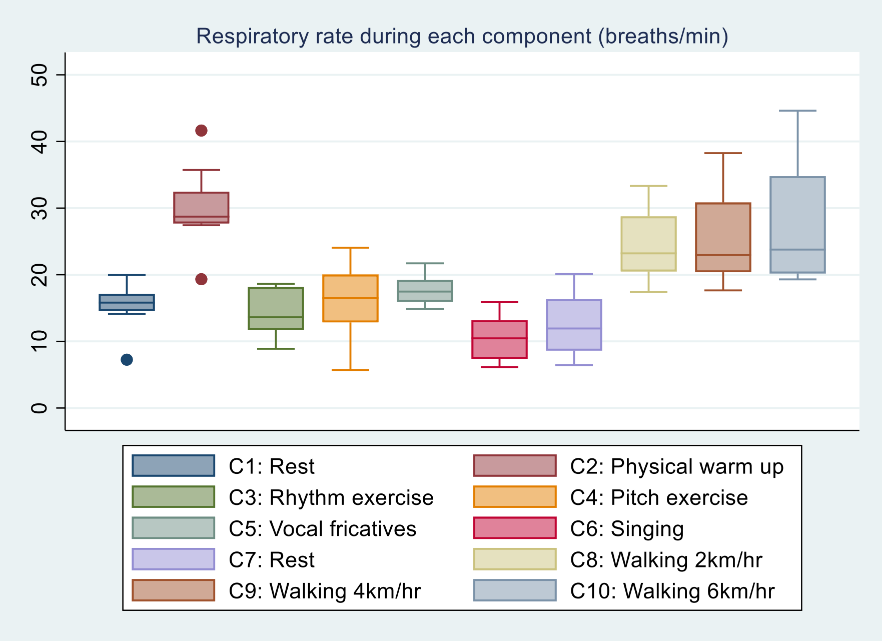

Supplement: Supplementary data [file bmjresp-2021-000959supp001.pdf]
